# Supplementary material for: An Innovative Method for BTEX Emission Inventory and Development of Mitigation Measures in Developing Countries—A Case Study: Ho Chi Minh City, Vietnam
Source: Int J Environ Res Public Health. 2022 Dec 2;19(23):16156. doi: 10.3390/ijerph192316156 (PMC9738250; doi:10.3390/ijerph192316156)
Supplement: Supplementary file 1 [file ijerph-19-16156-s001.zip › ijerph-2002639-supplementary.pdf]

Table S1 Number of questionnaires during the campaign

	Moto	Car	LDV	Bus	HDV	Total
Ho' study (Bang & Clappier, 2011)*	718	610	523	576	497	**2,924**
This study	385	193	30	81	30	**770**
**Total**	**1,103**	**803**	**553**	**657**	**527**	**3,694**

* Bang, H. Q. & Clappier, A. (2011). Road traffic emission inventory for air quality modeling and to evaluate the abatement strategies: A case of Ho Chi Minh city, Vietnam. *Atmos Environ.*, *45*.

Table S2. The information of flight data (trips/year)

Types	Domestic flight	International flight	Total	Types	Domestic flight	International flight	Total
A321	3,0145	10,763	40,908	A319	-	252	252
A320	36,325	7,787	44,112	E90	-	276	276
ATR72	2,920	1,470	4,390	A318	-	288	288
A330	2,248	1,858	4,105	A359	-	437	437
A350	1,460	2,403	3,863	B77W	-	183	183
ATR7	2,920	-	2,920	B788	-	183	183
B787	2,190	168	2,358	B735	-	91	91
B737	2,373	2,715	5,088	DH8	-	151	151
B763	-	730	730	B772	-	36	36
B738	-	1,794	1,794	B777	-	880	880
Airbus A300-600	-	1,056	1,056	Boeing B747	-	1,008	1,008
Boeing B757	-	144	144	Boeing B767	-	480	480
Boeing B777	-	736	736	MD11	-	936	936
